# Inside a Killer: Immune Signals May Promote Vascular Growth

**DOI:** 10.1371/journal.pbio.0040030

**Published:** 2005-12-27

“Natural killer cells”—their very name chillingly evokes their primary function as the hit men of the body's immune system. But according to a new study by Sumati Rajagopalan, Eric Long, and their colleagues, these killers may also play a part in ensuring the success of a pregnancy. And the signal that provokes this unexpected action is itself unusual, acting not from the surface of the cell, but from within it.

Natural killer (NK) cells are a type of white blood cell. NK cells circulate in the blood stream and are also residents in a few tissues, including the uterus, where they are the primary type of white blood cell. On their surfaces, they carry a variety of receptors, which sense the exterior environment and trigger a host of internal responses. When activated, NK cells are cytotoxic—they kill target cells, such as virus-infected cells and tumor cells—and secrete soluble signaling molecules that help mount immune responses to infection by parasites, bacteria, and other invaders.

NK cell receptors bind to proteins on the surfaces of other cells. One type of NK cell receptor, called killer cell immunoglobulin-like receptor (KIR)2DL4, binds to the protein human leukocyte antigen (HLA)-G, whose precise role is unknown and which occurs in both membrane-bound and secreted (soluble) forms. Interestingly, HLA-G is primarily found on trophoblast cells, a type of embryonic tissue that invades the uterine lining to support the developing fetus. [Fig pbio-0040030-g001]

**Figure pbio-0040030-g001:**
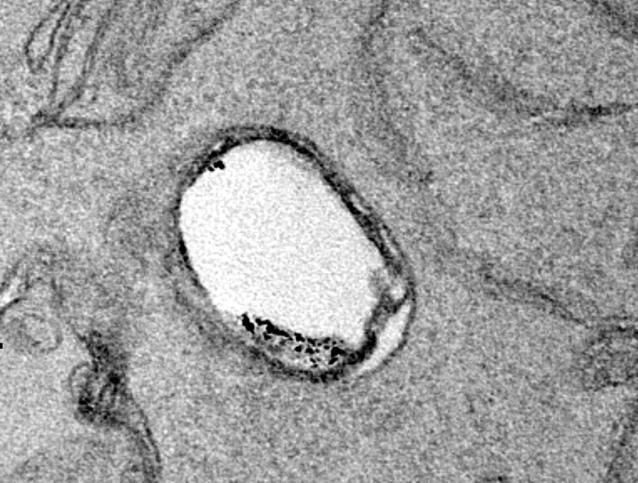
A natural killer cell receptor called KIR2DL4 stimulates an immune signaling pathway from inside an intracellular vesicular structure (shown above)

The response of NK cells activated by KIR2DL4 is unusual: they release cytokines, immune chemicals that trigger actions in other cells. But this cytokine release, unlike that associated with other receptors, is not accompanied by cytotoxicity.

To understand the role of KIR2DL4-bearing NK cells in the uterus, and how they might respond to HLA-G signals, the authors examined the KIR2DL4/HLA-G interaction in detail. By activating the receptor with an antibody (to ensure that only KIR2DL4 would be triggered), they first showed that cytokine secretion could be triggered only when the antibody was soluble, not when bound to a solid surface. This behavior immediately suggested that the interaction between the two doesn't end at the membrane, since if it did, the immobilized antibodies would have sufficed to activate the cells.

They next showed that KIR2DL4 is brought into the cell by endocytosis, an energy-requiring process that cells use to carry membrane-bound molecules to the interior. They found that KIR2DL4 was present in endosomes, the vesicles formed by endocytosis. KIR2DL4 was found primarily in so-called early endosomes, but much less so in later stages, when the vesicle is being prepared for merging with a lysosome, the cell's disposal system.

What is KIR2DL4 doing in the early endosome? Rajagopalan et al. found that the receptor binds to the soluble, secreted form of HLA-G, and that HLA-G is carried with it into the endosome. Here, the complex triggered cytokine production and NK cell signaling. KIR2DL4 that could not reach the endosome could not trigger cytokine production. Therefore, the authors suggest that KIR2DL4 signaling happens from within the endosome, not at the cell surface. The cytokines produced are known to be active not only in inflammatory reactions but also in angiogenesis, or formation of new blood vessels.

Endosomes have emerged recently as important signaling compartments, and these results support and extend that understanding. On the cell surface, the receptor senses the environment for soluble ligand; once internalized into endosomes, the membrane-bound receptor interacts with signaling molecules within endosomes to set off a cascade of reactions, ultimately leading to protein production. The authors suggest that internal signaling may increase the fidelity of the desired response by avoiding the many possibly conflicting signals arising from other receptors at the surface during cell–cell interactions.

The results presented here also suggest some intriguing ideas regarding how the immune system responds to the developing embryo. Within the uterus, the embryo represents a special challenge, since it is composed of cells bearing partly foreign (that is, paternal) genetic material that must not only be tolerated but allowed to intimately intertwine with the mother's tissue to develop a new blood supply. This remodeling of the vascular system is partly under immune system control. The angiogenic cytokines triggered by KIR2DL4 activation may support this process.

Further support for this idea comes from recent observations that a higher level of soluble HLA-G promotes higher rates of successful pregnancy, while reduced HLA-G correlates with higher rates of preeclampsia, a condition caused by insufficient vascular remodeling during pregnancy. Additional study of the KIR2DL4/ HLA-G signaling pathway may lead to a better understanding of this potentially fatal complication of pregnancy, and better ways to prevent it. As the production of soluble HLA-G can also be induced in certain cell types, including tumor cells, the KIR2DL4/HLA-G signaling pathway may also serve additional functions, unrelated to pregnancy. *—Richard Robinson*

